# Impact of Mild COVID-19 History on Oral-Gut Microbiota and Serum Metabolomics in Adult Patients with Crohn’s Disease: Potential Beneficial Effects

**DOI:** 10.3390/biomedicines12092103

**Published:** 2024-09-14

**Authors:** Bingjie Xiang, Qi Zhang, Huibo Wu, Jue Lin, Zhaoyuan Xu, Min Zhang, Lixin Zhu, Jun Hu, Min Zhi

**Affiliations:** 1 Department of Gastroenterology, The Sixth Affiliated Hospital, Sun Yat-sen University, Guangzhou 510655, China; xiangbj3@mail2.sysu.edu.cn (B.X.); zhangq233@mail2.sysu.edu.cn (Q.Z.); wuhb6@mail2.sysu.edu.cn (H.W.); linj277@mail2.sysu.edu.cn (J.L.); xuzhy57@mail2.sysu.edu.cn (Z.X.); zhangm72@mail.sysu.edu.cn (M.Z.); 2 Guangdong Institute of Gastroenterology, Guangzhou 510655, China; zhulx6@mail.sysu.edu.cn; 3 Guangdong Provincial Key Laboratory of Colorectal and Pelvic Floor Disease, The Sixth Affiliated Hospital, Sun Yat-sen University, Guangzhou 510655, China; 4 Key Laboratory of Human Microbiome and Chronic Diseases, Sun Yat-sen University, Ministry of Education, Guangzhou 510655, China; 5 Department of Colorectal Surgery, The Sixth Affiliated Hospital, Sun Yat-sen University, Guangzhou 510655, China; 6 Biomedical Innovation Center, The Sixth Affiliated Hospital, Sun Yat-sen University, Guangzhou 510655, China

**Keywords:** Crohn’s disease, COVID-19, oral microbiota, gut microbiota, metabolome, inflammatory bowel disease

## Abstract

The impact of coronavirus disease 2019 (COVID-19) history on Crohn’s disease (CD) is unknown. This investigation aimed to examine the effect of COVID-19 history on the disease course, oral-gut microbiota, and serum metabolomics in patients with CD. In this study, oral-gut microbiota and serum metabolomic profiles in 30 patients with CD and a history of mild COVID-19 (positive group, PG), 30 patients with CD without COVID-19 history (negative group, NG), and 60 healthy controls (HC) were assessed using 16S rDNA sequencing and targeted metabolomics. During follow-up, the CD activity index showed a stronger decrease in the PG than in the NG (*p* = 0.0496). PG patients demonstrated higher α-diversity and distinct β-diversity clustering in both salivary and fecal microbiota compared to NG and HC individuals. Notably, the gut microbiota composition in the PG patients showed a significantly greater similarity to that of HC than NG individuals. The interaction between oral and intestinal microbiota in the PG was reduced. Moreover, serum metabolome analysis revealed significantly increased anti-inflammatory metabolites, including short-chain fatty acids and N-Acetylserotonin, among PG patients; meanwhile, inflammation-related metabolites such as arachidonic acid were significantly reduced in this group. Our data suggest that the gut microbiota mediates a potential beneficial effect of a mild COVID-19 history in CD patients.

## 1. Introduction

The global coronavirus disease (COVID-19) pandemic, caused by severe acute respiratory syndrome coronavirus 2 (SARS-CoV-2) outbreaks in 2019, has substantially impacted global health [1]. While vaccination and public health control measures effectively manage its spread, eliminating the virus is challenging. It appears that SARS-CoV-2 will remain alongside humans for an extended period. Amid the expanding COVID-19 pandemic, there is increasing public concern regarding its impact on individuals with chronic conditions, such as Crohn’s disease (CD). CD, a subtype of inflammatory bowel disease (IBD), is a chronic, recurrent, and incurable inflammatory condition that affects the digestive tract, often accompanied by extraintestinal manifestations [2,3]. The occurrence of COVID-19 in patients with IBD is comparable to that in the general population [4]. SARS-CoV-2, detectable in stools even after respiratory samples test negative, raises concerns about the possible long-term gastrointestinal impact [5]. Many researchers have hypothesized, based on theoretical mechanisms and pathway theories, that COVID-19 could act as a risk factor for either the onset or relapse of IBD. These hypotheses are rooted in the inflammatory and immune responses triggered by the virus, which could theoretically exacerbate existing IBD conditions or unmask new cases [6,7]. However, despite these theories, evidence remains lacking to definitively support the notion that COVID-19 directly leads to the onset or exacerbation of IBD. Therefore, further research is required to investigate the effect of COVID-19 on individuals with CD.

Alterations in the oral and gut microbiota composition in patients with COVID-19 were observed at diagnosis, during the disease course, and in the follow-up period, in contrast to individuals without COVID-19 [8,9]. Significantly decreased α diversity was noted in the oral and gut microbiota of patients with COVID-19 [8]. Compared with healthy controls (HC), patients with COVID-19 exhibited a reduction in butyric acid-producing bacteria and an increase in lipopolysaccharide-producing bacteria [9]. Compared with seasonal flu patients, those with COVID-19 displayed a significant increase in opportunistic pathogens, such as *Streptococcus* and *Fusobacterium* [10]. Furthermore, compared to patients with H1N1 influenza infection, those with COVID-19 showed a significantly higher level of Actinobacteria and Firmicutes phyla [11]. The abnormal alteration of oral-gut microbiota persisted for up to 1 year post SARS-CoV-2 [8] infection. COVID-19 typically lasts only a few days or weeks; nevertheless, its impact on the microbiota is more enduring. The crucial role of the oral gut microbiota and their metabolites in developing, progressing, and treating CD has been convincingly demonstrated. In cases of IBD, the gut microbiota is characterized by increased instability, reduced microbial diversity, elevated levels of Proteobacteria taxa, and decreased Firmicutes [12]. Notably, ectopic gut colonization by oral microbes is a distinctive feature of IBD, and the abnormal ectopic location of oral pathogens in the intestine exacerbates gastrointestinal inflammation [13,14]. Host metabolism is significantly affected by COVID-19. Lipid alterations in recovered patients of COVID-19 have been observed. In these patients, 122 lipid molecules exhibited an increase, while 47 lipid molecules showed a decrease [9]. Sphingosine-1-phosphate (S1P), closely tied to the inflammatory storm in COVID-19, had lower levels, indicating a worse prognosis [8]. S1P reached its lowest level during the infection period and rose gradually during recovery [8]. However, the metabolites and oral-gut microbiota composition in vulnerable patients with CD post-COVID-19 remain unclear.

Thus, we aimed to investigate the impact of COVID-19 history on the disease course, the oral-gut microbiota, and metabolites in patients with CD. Our data indicate an interplay between the metabolome and microbiota that may influence the disease course of CD in patients with a history of COVID-19. These results suggest that the gut microbiota mediates a potential beneficial effect of COVID-19 history in patients with CD.

## 2. Materials and Methods

### 2.1. Study Design

This study continuously enrolled 60 CD patients who visited the Sixth Affiliated Hospital of Sun Yat-sen University from November 2022 to May 2023 and consented to participate, and 60 healthy individuals who participated from June 2018 to November 2019. Fecal and saliva samples were collected from all participants. Based on their history of COVID-19, patients with CD were divided into two groups: 30 with a history of mild infection (positive group, PG) and 30 without a history of infection (negative group, NG). The CD activity index (CDAI) was assessed by clinical experts during sample collection. The patients in the PG and NG groups were further classified into subgroups: the active group (CDAI ≥ 150), which encompassed mild (CDAI = 150–220), moderate (CDAI = 221–450), and severe (CDAI > 450) disease activity, and the remission group (CDAI < 150) [15]. Demographic and clinical data such as sex, age, body mass index (BMI), medication use, and location/behavior were obtained from medical records. In addition, the CDAI of the patients with CD was assessed again 6 and 9 months after sample collection by clinical experts. 

### 2.2. Eligibility Criteria

The inclusion criteria were the following: (1) CD diagnosed by experienced clinicians based on clinical, pathological, endoscopic, laboratory, and radiological features [2,3]; (2) COVID-19 diagnosis determined based on the positive PCR or antigen test [16,17]; (3) HC free of both CD and COVID-19; (4) age of all participants: 18–75 years; (5) PG participants all with mild COVID-19. Mild COVID-19 was defined as showing mild symptoms such as fever, cough, and sore throat, without pneumonia symptoms and pneumonia imaging evidence according to the WHO interim guidance (Clinical management of COVID-19: interim guidance, 27 May 2020); (6) For COVID-19 infection, the PG group only received symptomatic treatment such as antipyretics and cough suppressants. 

The exclusion criteria were the following: (1) use of antibiotics, probiotics, or proton pump inhibitors within 1 month before sample collection; (2) pregnancy; (3) gastrointestinal cancer, tuberculosis, or Behcet’s disease; (4) gastrointestinal resection.

### 2.3. Outcomes

The outcomes of interest were the following: (1) clinical symptom improvement, i.e., changes in CDAI; (2) composition of the microbiota in the PG group compared to the HC and NG groups; (3) composition of serum metabolic groups in the PG group compared to the NG group.

### 2.4. Sample Collection

Fresh fecal samples were collected in sterile tubes immediately after defecation, divided into aliquots (200 mg), and promptly stored at −80 °C until needed. Saliva samples were collected in sterile containers, mixed with an equal volume of DNA stabilization buffer, and stored at −80 °C until use. The DNA stabilization buffer, totaling 250 mL, contained 1.461 g NaCl, 2.5 mL Tris HCl, 5 mL 0.5 M EDTA, 12.5 mL 10% SDS, 2.5 mL 20 mg/mL Proteinase K Solution, and 227.5 mL sterilized water. All participants were instructed to avoid oral hygiene procedures overnight and refrain from drinking or eating for at least 2 h before saliva collection. Simultaneously, peripheral blood was harvested and centrifuged at 3000 rpm for 10 min, and the serum was divided into aliquots (100 μL) and stored at −80 °C.

### 2.5. DNA Extraction and High-Throughput Sequencing

Microbial genomic DNA (gDNA) was extracted from samples using a stool DNA Kit (Magen, Guangzhou, China; cat. D3141B) according to the manufacturer’s instructions. Saliva DNA extraction was performed using a saliva DNA Kit (Magen, Guangzhou, China; cat. D3134). The V5–V6 hypervariable region of bacterial 16S rDNA was amplified from gDNA using the forward primer 784F (5′-RGGATTAGATACCC-3′) and the reverse primer 1064R (5′-CGACRRCCATGCANCACCT-3′). Subsequently, the PCR products were purified and subjected to paired-end sequencing using a high-throughput sequencer (MGISEQ2000, BGI, Shenzhen, China) following standard protocols. The raw data were quality-filtered and spliced using FLASH (v1.2.11). The DADA2 in QIIME2 was utilized for denoising to obtain amplicon sequence variants (ASVs)—which are sequences with 100% similarity—and to generate the feature table. For the alpha diversity analysis, we calculated the Chao index. For the beta diversity analysis, principal coordinate analysis (PCoA) was performed using the R language. The Wilcoxon test was utilized to assess significantly different bacteria, with a *p*-value and false discovery rate (FDR) < 0.05. Network correlation analysis was based on Spearman’s correlation (|correlation coefficient| > 0.6, *p* < 0.05) generated using R (version 4.3.1). The resulting networks were subsequently processed and visualized using Gephi software (version 0.9.2).

### 2.6. Metabolomic Data Generation and Analysis

Serum samples were submitted to Metabo-Profile Biotechnology (Shanghai, China) Co., Ltd. for metabolomics detection. The quantification of targeted metabolites was performed using an ultra-performance liquid chromatography coupled with a tandem mass spectrometry (UPLC-MS/MS) system (ACQUITY UPLC-Xevo TQ-S, Waters Corp., Milford, MA, USA). All standards for the targeted metabolites were prepared at a concentration of 5.0 mg/mL. Dots exceeding ± 3 standard deviations in score values are generally considered potential outliers. If improper sample preparation procedures or injections are identified, outlying samples would be re-prepared and reanalyzed. Raw data were processed using TMBQ software (v1.0, Metabo-Profile, Shanghai, China). Statistical analyses, including univariate, multivariate, and pathway enrichment analyses, were conducted using the iMAP platform (v1.0, Metabo-Profile, Shanghai, China). 

### 2.7. Statistical Analysis

Descriptive data are presented as mean ± SD or median (25–75 interquartile range). To assess differences in taxonomic abundance between groups, students’ *t*-tests or Wilcoxon tests were conducted. Chi-square and Fisher’s exact tests were performed using SPSS, version 26.0.

## 3. Results

### 3.1. Participant Characteristics

**Figure 1:**
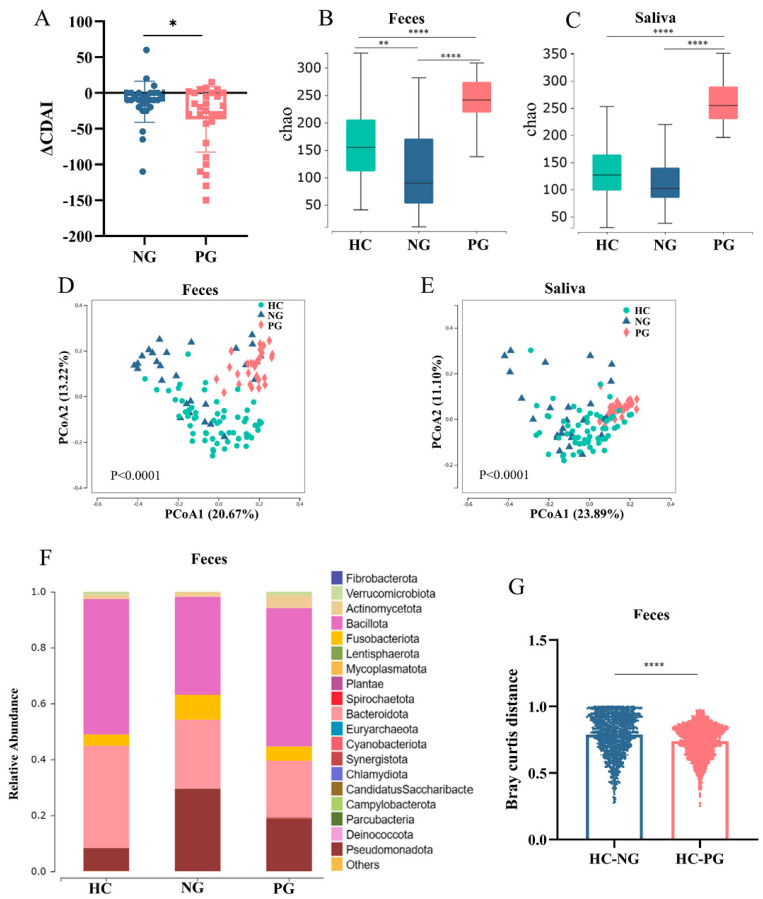
Recovery of clinical activities and microbial community. (**A**) CDAI changes from the initial enrollment to the 6-month follow-up. (**B**,**C**) Differences of α-diversities. (**D**,**E**) β-diversities calculated using UniFrac-based unweighted principal coordinate analysis (PCoA). (**F**) Relative abundance of bacterial phyla. (**G**) Bray Curtis distance of gut microbiota between HC and NG or PG. CDAI, Crohn’s disease activity index; HC, healthy control; NG, negative group; PG, positive group. * *p* < 0.05; ** *p* < 0.01; **** *p* < 0.0001.

Table 1 displays the demographic and clinical characteristics of the participants. No significant differences were observed in sex (HC: 76.7% male, NG: 66.7% male, PG: 56.7% male) and age (HC: 21.8 [19.56–24.5], NG: 30.37 ± 8.65, PG: 33.13 ± 7.80) among the three groups. Similarly, the PG and NG did not significantly differ in BMI (NG: 18.77 ± 3.35, PG: 20.24 ± 2.82), staging (NG: 73.3% active stage, PG: 73.3% active stage), lesion location (NG: 66.7% ileocolon, PG: 56.7% ileocolon), and other factors. All individuals in the PG recovered from COVID-19 within 7–14 days, and the samples were collected 2–4 months after recovering from COVID-19. CDAI was assessed at the time of enrollment and again after 6 and 9 months; the results showed a more pronounced CDAI decline (ΔCDAI) in the PG than NG (Figure 1A and Figure S1).

### 3.2. Altered Oral and Gut Microbiota Composition in COVID-19-Infected Patients with CD

A total of 176 samples, consisting of 88 saliva and 88 fecal samples, underwent 16S rDNA gene sequencing, revealing 23 phyla, 39 classes, 73 orders, 136 families, 424 genera, and 897 species. The diversity of microbial communities within (α diversity) and between (β diversity) samples in the three groups was analyzed at the ASV level. Alpha analyses revealed that the PG had significantly higher oral (HC: 127.0 [98.0–165.8], NG: 102.0 [84.5–199.3], PG: 256.0 [227.8–305.8]; HC vs. PG: *p* < 0.0001, NG vs. PG: *p* < 0.0001) and fecal (HC: 155.0 [111.3–209.8], NG: 89.5 [50.75–181.0], PG: 241.5 [18.3–277.3]; HC vs. PG: *p* < 0.0001, NG vs. PG: *p* < 0.0001) microbial diversity than the other groups based on the Chao index, indicating that COVID-19 substantially altered the diversity and richness of the oral and gut microbiota (Figure 1B,C). Regarding β diversity, principal coordinate analysis (PCoA) based on unweighted UniFrac distance was conducted to compare microbial communities among the three groups, revealing significant differences (saliva: *p* < 0.0001; feces: *p* < 0.0001) (Figure 1D,E). The lower β diversity indicated smaller heterogenicity in the microbial communities of the PG.

**Figure 2:**
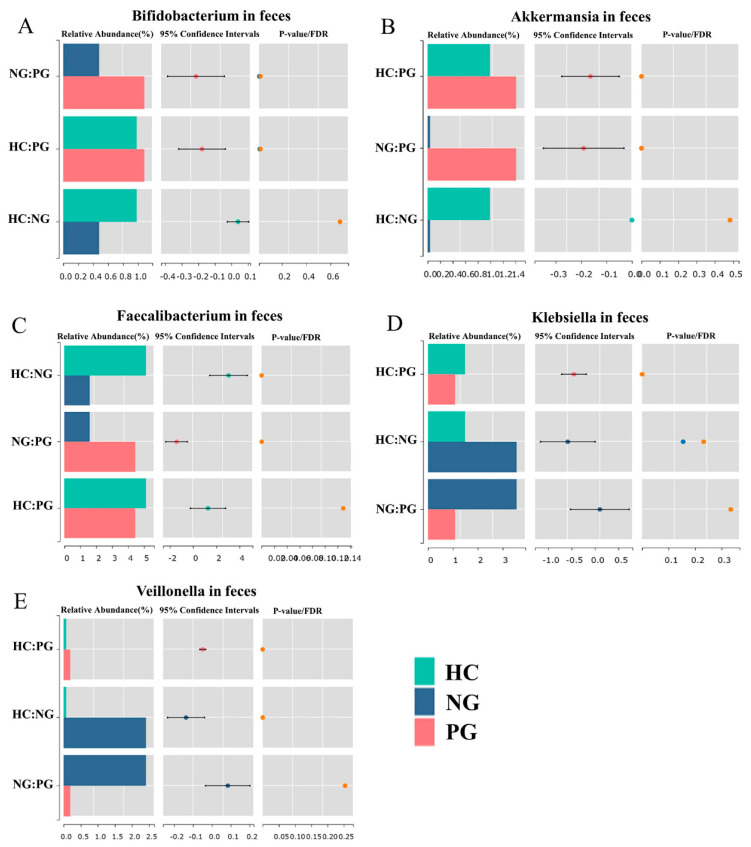
Relative abundance of genera *Bifidobacterium* (**A**), *Akkermansia* (**B**), *Faecalibacterium* (**C**), *Klebsiella* (**D**)*,* and *Veillonella* (**E**) in three groups.

Discernible shifts have been observed in gut microbiota compositions in the PG according to the stacked bar chart (Figure 1F). Specifically, the gut microbiota in the PG was characterized by an increase in the Bacillota phylum and a decrease in the Fusobacteriota and Pseudomonadota phyla, approaching levels similar to those in the HC (Figure 1F). At the genus level, the gut microbiota in the PG was characterized by an increase in *Megamonas*, *Faecalibacterium*, *Akkermansia*, and *Phocaeicola*, and a decrease in *Fusobacterium*, *Bacteroides*, and *Veillonella*, approaching levels similar to those in the HC (Figure S2A). At the species level, the gut microbiota in the PG was characterized by an increase in *Akkermansia muciniphila, Ruminococcoides bili, Megamonas funiformis, Phocaeicola dorei,* and *Faecalibacterium duncaniae*, and a decrease in *Fusobaccterium varium, Klebsiella pneumoniae,* and *Veillonella dispar*, approaching levels similar to those in the HC (Figure S2B). The Bray–Curtis distance between the PG and HC (0.81 [0.69–0.92]) was significantly lower than that between the NG and HC (0.76 [0.66–0.83]), indicating that the gut microbiota in the PG was more similar to that in the HC group than in the NG (Figure 1G). In the gut, three genera that acted as probiotics—*Bifidobacterium* (NG: 0.48 ± 1.10%, PG: 1.08 ± 2.76%; *p* = 0.0196 and FDR = 0.0491), *Akkermansia* (NG: 0.04 ± 0.10%, PG: 1.41 ± 4.66%; *p* = 0.0001 and FDR < 0.0001)*,* and *Faecalibacterium* (NG: 1.59 ± 3.42%, PG: 4.44 ± 6.94%; *p* < 0.0001 and FDR < 0.0001)—were significantly enriched in the PG compared to the NG (Figure 2A–C). Conversely, the opportunistic pathogens *Klebsiella* (NG: 3.56 ± 9.07%, PG: 1.09 ± 1.12%; *p* = 0.0005 and FDR = 0.0020) and *Veillonella* (NG: 2.41 ± 7.58%, PG: 0.19 ± 0.29%; *p* < 0.0001 and FDR < 0.0001) were enriched in the NG (Figure 2D,E).

Additionally, in the remission and active subgroups, the α diversity of oral (HC vs. PG: *p* = 0.0008, NG vs. PG: *p* = 0.0221) and gut (HC vs. PG: *p* = 0.0034, NG vs. PG: *p* = 0.0082) microbiota was significantly increased in the PG based on the Chao index (Figure S3A–D). Regarding β diversity, significant differences were observed among the groups (saliva in remission: *p* = 0.0084; feces in remission: *p* < 0.0001; saliva in active: *p* < 0.0001; feces in active: *p* < 0.0001) (Figure S3E–H). No significantly different genus was observed during the remission stage between the PG and NG. In the active stage, there were 31 different salivary genera and 39 different fecal genera between the PG and the NG. This suggested that COVID-19 had a more pronounced impact on the microbiota of patients with active CD. 

### 3.3. Interaction between the Oral and Gut Microbial Communities

**Figure 3:**
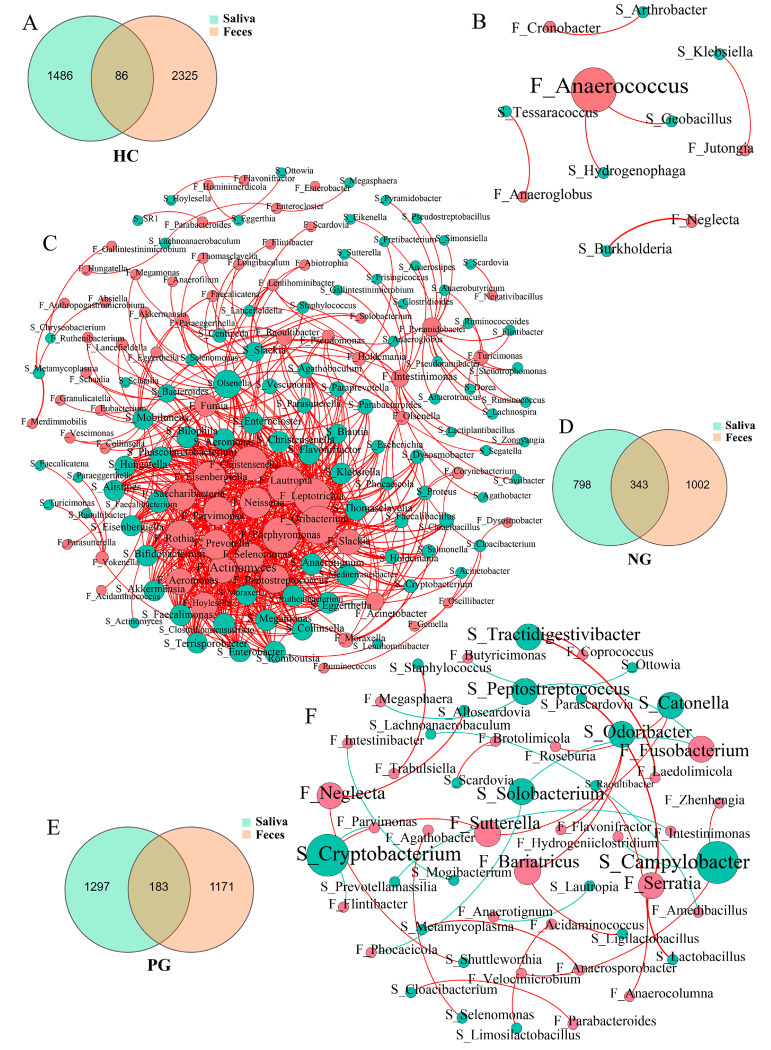
Interaction between oral and gut microbiota. (**A**) Venn diagram illustrating ASVs of oral and gut microbiota in HC. (**B**) Spearman’s correlation network between oral and gut microbiota in HC. (**C**) Venn diagram illustrating ASVs of oral and gut microbiota in NG. (**D**) Spearman’s correlation network between oral and gut microbiota in NG. (**E**) Venn diagram illustrating ASVs of oral and gut microbiota in PG. (**F**) Spearman’s correlation network between oral and gut microbiota in PG. The red circle represents fecal microbiota, and the blue circle represents salivary microbiota. The size of the circles represents the quantity of significant correlation relationships. The red line represents positive correlation. The blue line represents negative correlation. F_, fecal microbiota; S_, salivary microbiota; HC, healthy control; NG, negative group; PG, positive group; ASV, amplicon sequence variants.

Saliva and feces shared 86 ASVs in HC, 343 in NG, and 183 in PG (Figure 3A,D,E). The Spearman’s correlation network depicting the association between the salivary and gut microbiota showed 6 significant relationships in HC, 730 in NG, and 33 in PG (Figure 3B,C,F). These results indicate that taxa within the NG exhibited elaborate and robust interrelationships compared to the other groups, while the oral-gut axis showed signs of recovery in the PG towards HC.

### 3.4. Metabolomic Changes in Participant Serum

**Figure 4:**
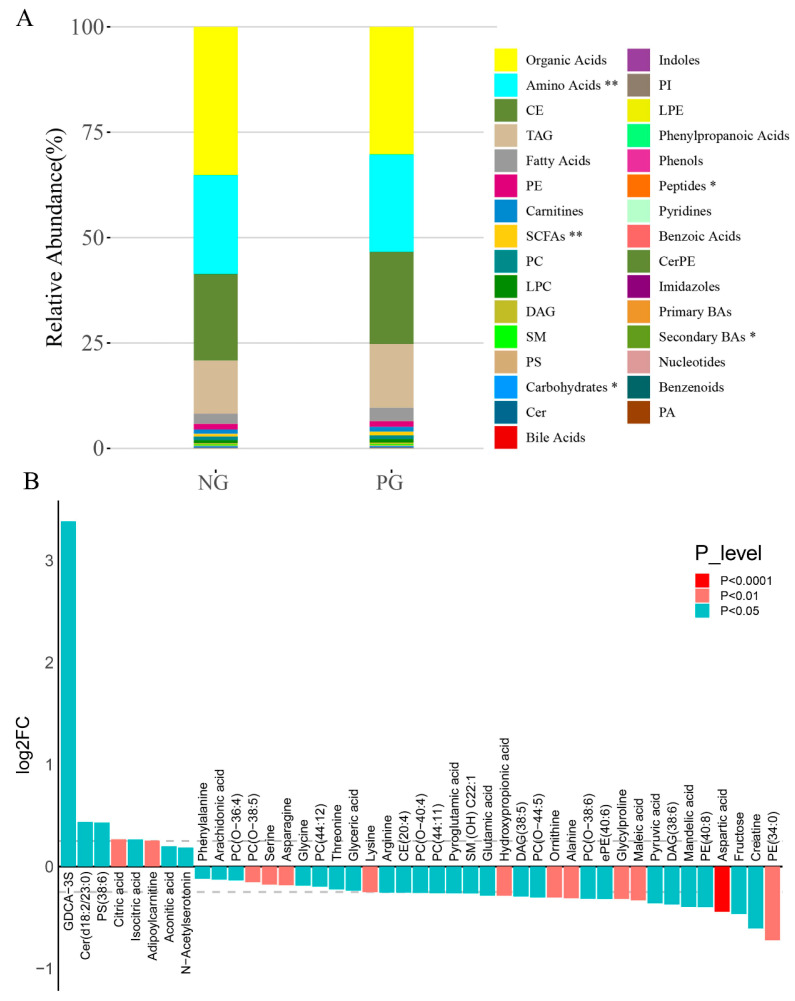
Serum metabolite composition. (**A**) Relative abundance of each metabolite class in the negative and positive groups; * *p* < 0.05; ** *p* < 0.01. (**B**) Significantly different metabolites (n = 43); log2FC > 0 represents an increase in the PG group, while a negative value indicates a decrease. PG, positive group.

**Figure 5:**
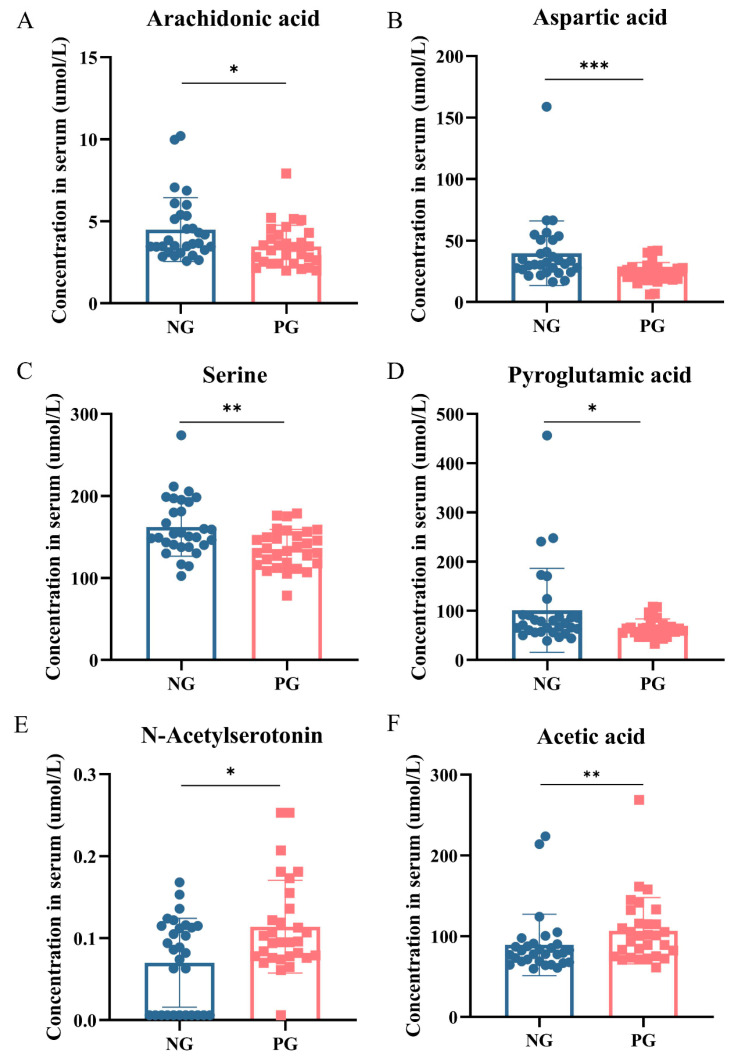
Boxplot of serum arachidonic acid (**A**), aspartic acid (**B**), serine (**C**), pyroglutamic acid (**D**), N−Acetylserotonin (**E**), and acetic acid (**F**) in the NG and PG. NG, negative group; PG, positive group. * *p* < 0.05; ** *p* < 0.01; *** *p* < 0.001.

Based on the differences in oral and gut microbiota composition between the PG and NG groups, we further investigated the serum metabolomics in the two groups. A total of 521 metabolites were detected, and the composition of their categories is shown in Figure S4: organic acids had the highest proportion (32.88%), followed by amino acids (23.27%), cholesterol esters (CE, 21.17%), triglycerides (TAG, 13.78%), and others. The relative abundance of each metabolite class in the PG and NG is illustrated in the stacked bar chart (Figure 4A). There were significant differences in amino acids (NG: 23.43%, PG: 23.08%), short-chain fatty acids (SCFAs) (NG: 0.67%, PG: 0.90%), carbohydrates (NG: 0.244%, PG: 0.238%), peptides (NG: 0.007%, PG: 0.006%), and secondary bile acids (NG: 0.001%, PG: 0.003%) between the PG and NG. Forty-three potential biomarkers were selected by combining the differential metabolites from univariate and multi-dimensional statistics, with a threshold value of variable importance in projection (VIP) >1 in multi-dimensional statistics and *p* < 0.05 and |log2FC| ≥ 0 in univariate statistics (Figure 4B). Among the 43 potential biomarkers, only 8 were increased in the PG, while the other 35 were decreased (Figure 4B). Notably, inflammation-related metabolites, including arachidonic acid (NG: 3.643 [3.212–5.347], PG: 3.332 [2.427–4.211], *p* = 0.0162), aspartic acid (NG: 31.68 [26.03–50.56], PG: 23.29 [19.04–27.74], *p* = 0.0001), serine (NG: 162.2 ± 35.66, PG: 135.5 ± 23.64, *p* = 0.0012), and pyroglutamic acid (NG: 72.83 [56.85–91.16], PG: 60.69 [48.70–71.75], *p* = 0.0415), were significantly reduced in PG, while N-acetylserotonin (NG: 0.084 [0.0061–0.1150], PG: 0.096 [0.0061–0.1408], *p* = 0.0321) and acetic acid (NG: 78.47 [68.48–90.34], PG: 100.7 [75.29–119.9], *p* = 0.0036), as anti-inflammatory metabolites, were significantly increased in PG (Figure 5A–F). Additionally, a substantial quantity of amino acids, including lysine, arginine, ornithine, glutamic acid, alanine, threonine, creatine, asparagine, phenylalanine, and glycine, were markedly decreased in the PG (Figure 4B). Pathway enrichment analysis was conducted using pathway-associated metabolite sets. Metabolic pathways were notably enriched, including glycine and serine metabolism, the urea cycle, ammonia recycling, and alanine metabolism (*p* < 0.05 and FDR < 0.05) (Figure S5).

### 3.5. Correlations between Microbiota and Serum Metabolites

**Figure 6:**
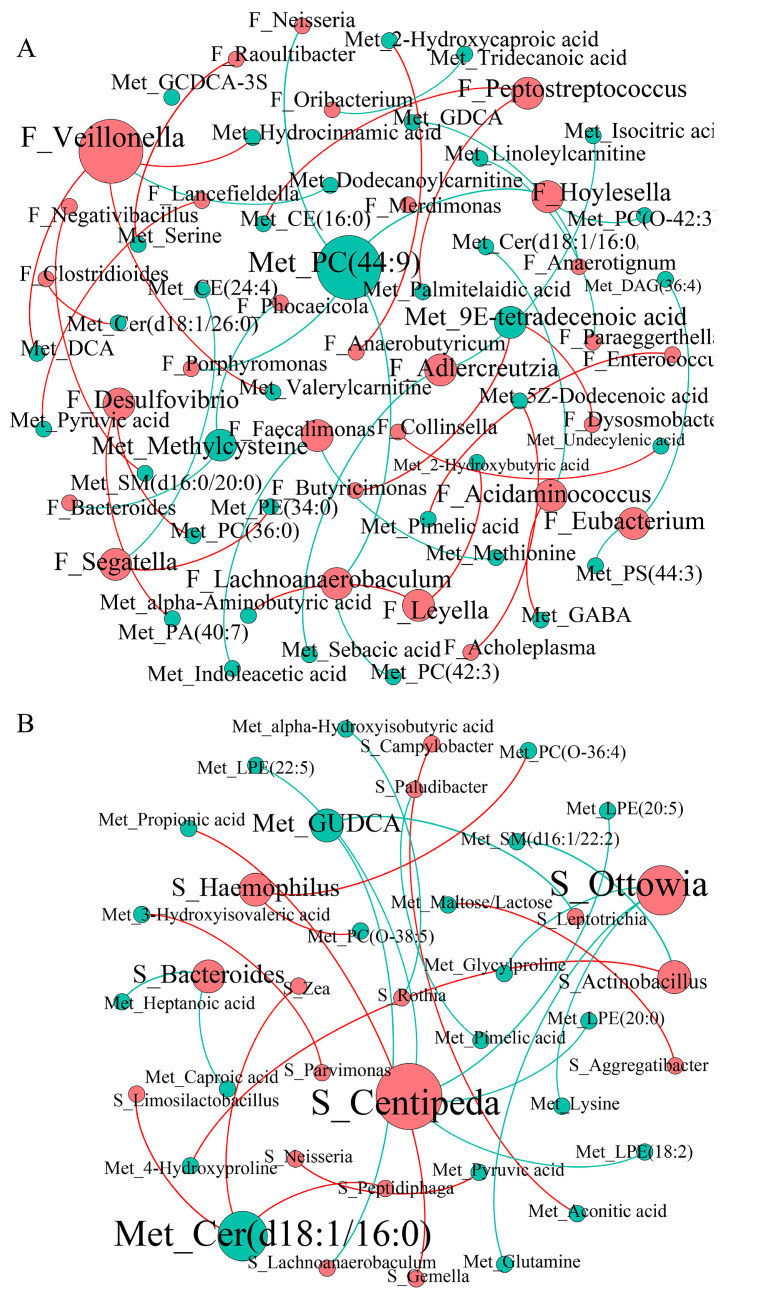
Interaction between oral-gut microbiota and serum metabolites. (**A**) Spearman’s correlation network between gut microbiota and serum metabolites in PG. (**B**) Spearman’s correlation network between oral microbiota and serum metabolites in PG. The red circle represents fecal or salivary microbiota, and the blue circle represents serum metabolites. The size of the circles represents the number of significant correlation relationships. The red line represents positive correlation, and the blue line represents negative correlation. F_, fecal microbiota; S_, salivary microbiota; PG, positive group.

We conducted a correlation analysis network to understand the potential association between the oral gut microbiota and metabolites. We identified many interconnected relationships between oral-gut microbiota and metabolites in the PG (Figure 6). Gut microbiota genera, including *Veillonella*, *Adlercreutzia*, *Segatella*, *Peptostreptococcus*, *Hoylesella*, *Desulfovibrio*, *Faecalimonas*, *Acidaminococcus*, *Eubacterium*, *Lachnoanaerobaculum,* and *Leyella*, showed a relatively high correlation with serum metabolites (Figure 6A). Saliva microbiota genera, including *Centipeda*, *Ottowia*, *Haemophilus*, *Bacteroides*, and *Actinobacillus*, strongly correlated with serum metabolites (Figure 6B).

## 4. Discussion

In this study, we found significant alterations in both oral and gut microbiota as well as serum metabolites in patients with CD and a history of COVID-19. The PG exhibited a higher diversity of microbes and distinct β diversity in their oral and gut microbiota compared to the NG. Interestingly, the composition of gut microbiota in patients with CD showed more similarity to that of HC. This finding highlights the dynamic recovery process of gut microbiota diversity among patients with CD following COVID-19 infection. 

In the PG, beneficial microbes were increased and harmful microbes decreased. Bacteria that acted as probiotics, such as *Bifidobacterium*, *Akkermansia*, and *Faecalibacterium*, were increased, while opportunistic pathogens, such as *Klebsiella* and *Veillonella*, decreased. These changes in microbiota may be associated with improved clinical outcomes in patients with CD in the PG compared to the NG. After the gut microbiota, the oral microbiota is considered the second most diverse microbial community within the human body. In healthy individuals, microbiota interactions in different ecological niches, such as oral and gut microbiota, are relatively infrequent [18,19]. However, previous studies have observed that patients with CD exhibited a greater similarity between their gut and oral microbiota than HC [19,20]. Consequently, it is hypothesized that oral bacteria may translocate to the gut and contribute to intestinal inflammation in pathological conditions such as CD [13,14,21]. In line with this notion, our study also revealed an increased interaction between oral and gut microbiota in patients with CD compared to HC. Notably, this phenomenon was more pronounced in the NG than in the PG, indicating that COVID-19 impeded abnormal crosstalk between oral and gut microbial communities.

Compared to NG, the metabolome was also found to be altered in the PG group. Most metabolites showed decreased levels in the PG group, consistent with a previous study indicating that patients with COVID-19 exhibit significant metabolic suppression compared to healthy individuals [22]. The inflammation-related metabolites were decreased in the PG group. Arachidonic acid—an unsaturated fatty acid released through the hydrolysis of membrane phospholipids by phospholipase—plays a crucial role as an inflammation mediator through its metabolites, such as prostaglandins, leukotrienes, and lipotoxins [23]. Serine is a central amino acid in the one-carbon metabolism network. The serine-dependent one-carbon metabolism plays a crucial role in T cell expansion [24] and the function of inflammatory macrophages [25]. It promotes the production of inflammatory factors such as interleukin-1β (IL-1β) and interferon [25,26]. Aspartic acid has been observed to correlate with CDAI and inflammatory cytokines levels and shows decreased levels in patients with active CD compared to those in remission [27]. Pyroglutamic acid levels were found to be elevated both in patients with IBD and rats with colitis [28,29,30], which has been associated with the upregulation of inflammatory factors and DNA damage [30]. Interestingly, these inflammation- or IBD-associated metabolites were observed at lower levels in PG than in NG; this suggests that COVID-19 infection might promote clinical improvement among patients with CD by downregulating these specific metabolites. In addition, the levels of anti-inflammatory metabolites such as N-acetylserotonin and acetic acid were increased in the PG. N-acetylserotonin, derived from tryptophan metabolism, possesses potent antioxidant and anti-inflammatory properties through receptors on innate and adaptive immune cells. It participates in the anti-inflammatory nuclear factor erythroid 2-related factor 2 (Nrf2)-heme oxygenase (HO)-1 pathway to ameliorate colitis [31,32]. N-Acetylserotonin was elevated in the PG compared to the NG, indicating that COVID-19 infection might promote clinical improvement in patients with CD by upregulating it. Consequently, COVID-19 might significantly impact both oral-gut microbiota and serum metabolome profiles. We further investigated the correlation between microbiota and serum metabolome in the PG, revealing significant associations between specific microbes and distinct metabolite levels. These findings provide evidence that COVID-19 might impact both oral-gut microbiota composition and serum metabolome profiles, highlighting their reciprocal influence on each other.

Our discoveries challenge preconceived notions regarding the adverse effects of COVID-19 and highlight its potential positive impact on microbial recovery. Tursi et al. [33] and Elbadry et al. [34] have identified that SARS-CoV-2 infection is a potential trigger factor for the de novo occurrence of IBD. However, it is important to note that the number of cases reported in these studies was relatively small (n = 6 and n = 2), which may limit the generalizability of their findings. In contrast, other researchers have observed no increase in the risk for de novo IBD following COVID-19 infection, suggesting that the relationship between SARS-CoV-2 and IBD may not be straightforward and could vary based on multiple factors [35]. Viganò et al. [36] observed an association between severe active IBD and COVID-19, suggesting that severe disease exacerbations might be linked to COVID-19 infection. However, they also noted that this association could be influenced by selection bias. Specifically, patients with severe IBD exacerbations who were hospitalized may have been more likely to be tested for COVID-19, potentially skewing the observed correlation. Additionally, they found that patients with IBD and COVID-19 reported more cases of diarrhea compared to patients with IBD without COVID-19 [36]. However, it was challenging to differentiate whether these gastrointestinal symptoms were primarily due to the digestive manifestations of COVID-19 or a result of IBD relapse or exacerbation. In contrast to these studies, our research focused on patients with CD who had experienced mild COVID-19 infections and had recovered for several months before the study. Our study specifically examined the impact of COVID-19 on existing CD rather than on new cases of IBD. Notably, our cohort did not include patients with severe active CD. By focusing on this specific population, our study aims to provide insights into how a mild, recovered COVID-19 infection affects pre-existing CD conditions. In agreement with our findings, previous studies have demonstrated that patients with COVID-19 with gastrointestinal symptoms exhibit significantly improved clinical outcomes [37,38]. This improvement has been linked to a notable reduction in inflammatory dendritic cells in the lamina propria of these patients [39]. Concurrently, RNA bulk sequencing demonstrated significant downregulation of the pathways related to antigen processing, Th17 cell differentiation, and IBD, indicating that SARS-CoV-2 infection in the intestines may alter the systemic immune cell landscape by shifting pro-inflammatory immune profiles towards a more favorable immune state [39]. This evidence suggests a plausible biological mechanism through which COVID-19 infection could influence the gut microbiome in patients with CD. Specifically, the reduction in pro-inflammatory immune responses and the downregulation of IBD-related pathways might contribute to an improved gut microbiome profile. The decreased circulating levels of critical inflammatory proteins, such as IL-6, IL-8, IL-17A, and CC chemokine ligand 28, observed by Livanos et al. [40], further indicate that COVID-19-induced changes in the immune system could enhance the gut microbiome environment by reducing overall inflammation.

This study has several limitations. First, the sample size was not sufficiently large, which may limit the generalizability of our findings. Second, we did not include patients with COVID-19 without CD in our analysis, which prevents drawing broader comparisons. Third, we lacked baseline microbiome and metabolome data, which could have provided a more comprehensive understanding of the differences between the PG and NG. Lastly, while the groups were followed up for several months concerning the CDAI, our study did not include longitudinal data on the microbiome and metabolome. This was primarily because most patients were from out of town, and follow-up was mainly conducted via phone, making it challenging to collect consistent longitudinal samples.

For future research, we recommend increasing the sample size to enhance the statistical power and generalizability of the findings. Including a broader cohort, such as patients with COVID-19 without CD, would provide more robust comparative data. Additionally, efforts should be made to establish a more comprehensive cohort that allows for the observation of dynamic changes across multiple time points, providing a deeper understanding of temporal evolution. Finally, exploring the underlying mechanisms driving these changes could offer significant insights into the interaction between CD, COVID-19, and the microbiome.

## 5. Conclusions

In conclusion, a history of mild COVID-19 is beneficial for patients with CD, which could be mediated by alterations in the gut microbiota. Specifically, patients with CD and a COVID-19 history displayed higher levels of bacteria that act as probiotics, lower levels of opportunistic pathogens, an increase in anti-inflammatory metabolites, and a decrease in inflammatory metabolites. 

## Figures and Tables

**Table 1:** Participant clinical characteristics.

Parameter	HC	NG	PG	*p*
Sex (M/F)	34/26	20/10	23/7	^a^ 0.567 ^b^ 0.069 ^c^ 0.494
Age (years)	21.8 (19.56–24.56) ^d^	30.37 ± 8.65 ^e^	33.13 ± 7.80	^a^ 0.199 ^b^ 0.543 ^c^ 0.114
BMI (kg/m^2^)	22.25 ± 3.51	18.77 ± 3.35	20.24 ± 2.82	^a^ 0.071 ^b^ 0.008 ^c^ 0.000
Duration (years)	-	3.32 ± 3.38	5.17 ± 4.65	0.08
Smoking	-	0	2	0.492
Biologics	- -	10 ^f^ 12.00 ± 9.15 ^g^	16 13.00 ± 9.28	0.192 0.79
Immunosuppressants	-	7 15.83 ± 8.23	3 18.00 ± 2.00	0.299 0.676
Glucocorticoids	-	3 6.00 ± 2.00	0 -	0.237 -
Enteral nutritional agent	-	3 5.83 ± 7.97	6 8.52 ± 15.26	0.472 0.79
5-ASA	-	7 6.83 ± 5.42	3 2.00 ± 1.73	0.299 1.88
Traditional Chinese medicine	-	0 -	2 10.00 ± 5.66	0.492 -
Non-use	-	7	1	0.052
CDAI < 150/CDAI ≥ 150	-	8/22	8/22	1.000
Mild/Moderate/Severe		14/8/0	15/7/0	1.000
Location (ileum/ileocolon/colon)	-	9/20/1	13/17/0	0.422
Stricturing	-	2	6	0.254
Penetrating	-	6	5	1.000
Stricturing and penetrating	-	1	1	1.000
Non-stricturing and non-penetrating	-	21	18	0.589

^a^ PG vs. NG; ^b^ PG vs. HC; ^c^ NG vs. HC; ^d^ median (25–75 interquartile range); ^e^ mean ± standard error of the mean; ^f^ number of patients who received the medication; ^g^ received treatment duration (month); BMI, body mass index; HC, healthy control group; NG, negative group; PG, positive group.

## Data Availability

The datasets used and analyzed in the present study are available from the corresponding author on reasonable request.

## Supplementary Materials

The following supporting information can be downloaded at: https://www.mdpi.com/article/10.3390/biomedicines12092103/s1, Figure S1: CDAI changes from the initial enrollment to the 6-month follow-up. Figure S2: Relative abundance of bacterial taxa at different taxonomic levels. (A) The relative abundance of bacterial genera. (B) The relative abundance of bacterial species; Figure S3: The diversities of oral and gut microbiota in active and quiescent CD. (A,B) The α-diversities of salivary and fecal microbiota in quiescent CD. (C,D) The α-diversities of salivary and fecal microbiota in active CD. (E,F) The β-diversities of salivary and fecal microbiota in quiescent CD. (G,H) The β-diversities of salivary and fecal microbiota in active CD; Figure S4: Pie chart showing the average abundance of metabolites across all samples; Figure S5: Pathway Enrichment Analysis Barplot Using Pathway-associated metabolite sets (Pathway-associated metabolite sets [SMPDB]).
