# Supplementary material for: Impact of Mild COVID-19 History on Oral-Gut Microbiota and Serum Metabolomics in Adult Patients with Crohn’s Disease: Potential Beneficial Effects
Source: Biomedicines. 2024 Sep 14;12(9):2103. doi: 10.3390/biomedicines12092103 (PMC11429124; doi:10.3390/biomedicines12092103)
Supplement: Supplementary file 1 [file biomedicines-12-02103-s001.zip › biomedicines-3176169-supplementary.pdf]

Supplemental Material

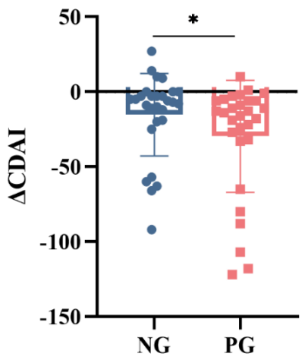

Figure S1. CDAI changes from the initial enrollment to the 6-month follow-up.

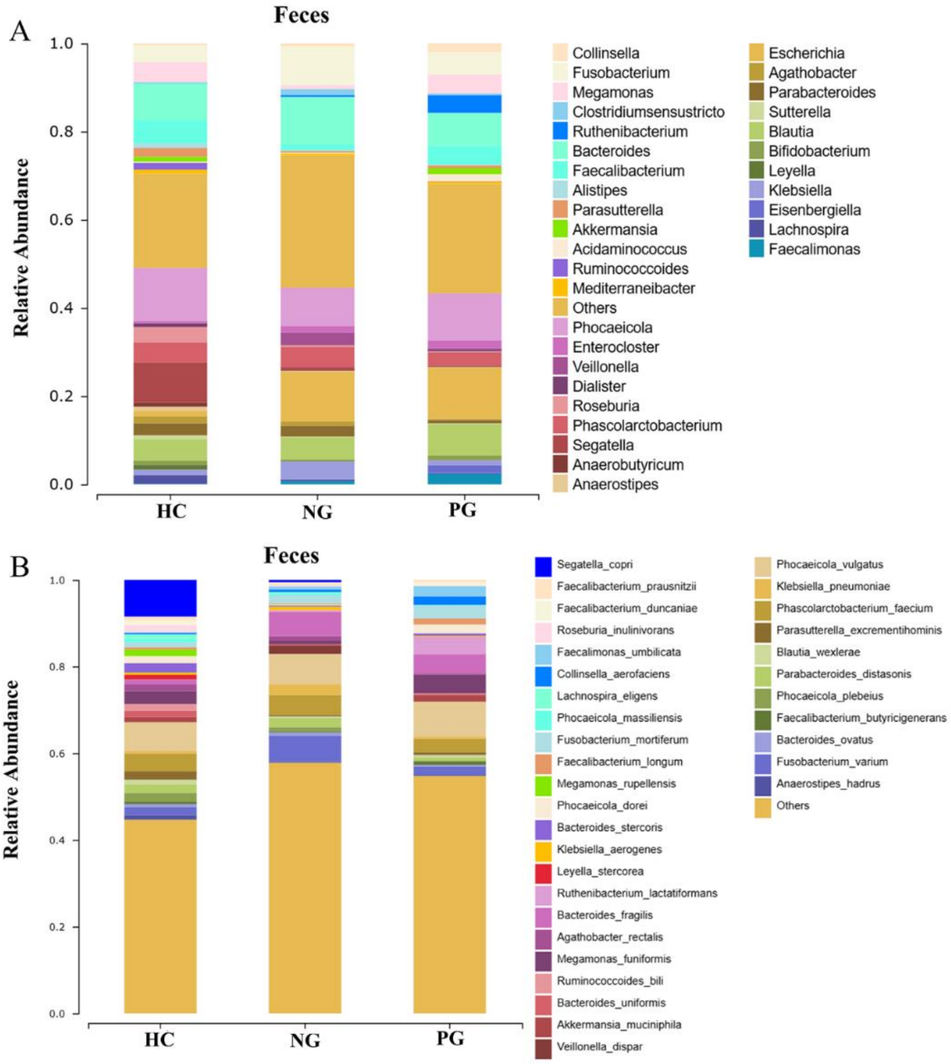

Figure S2. Relative abundance of bacterial taxa at different taxonomic levels. (A) The relative abundance of bacterial genera. (B) The relative abundance of bacterial species.

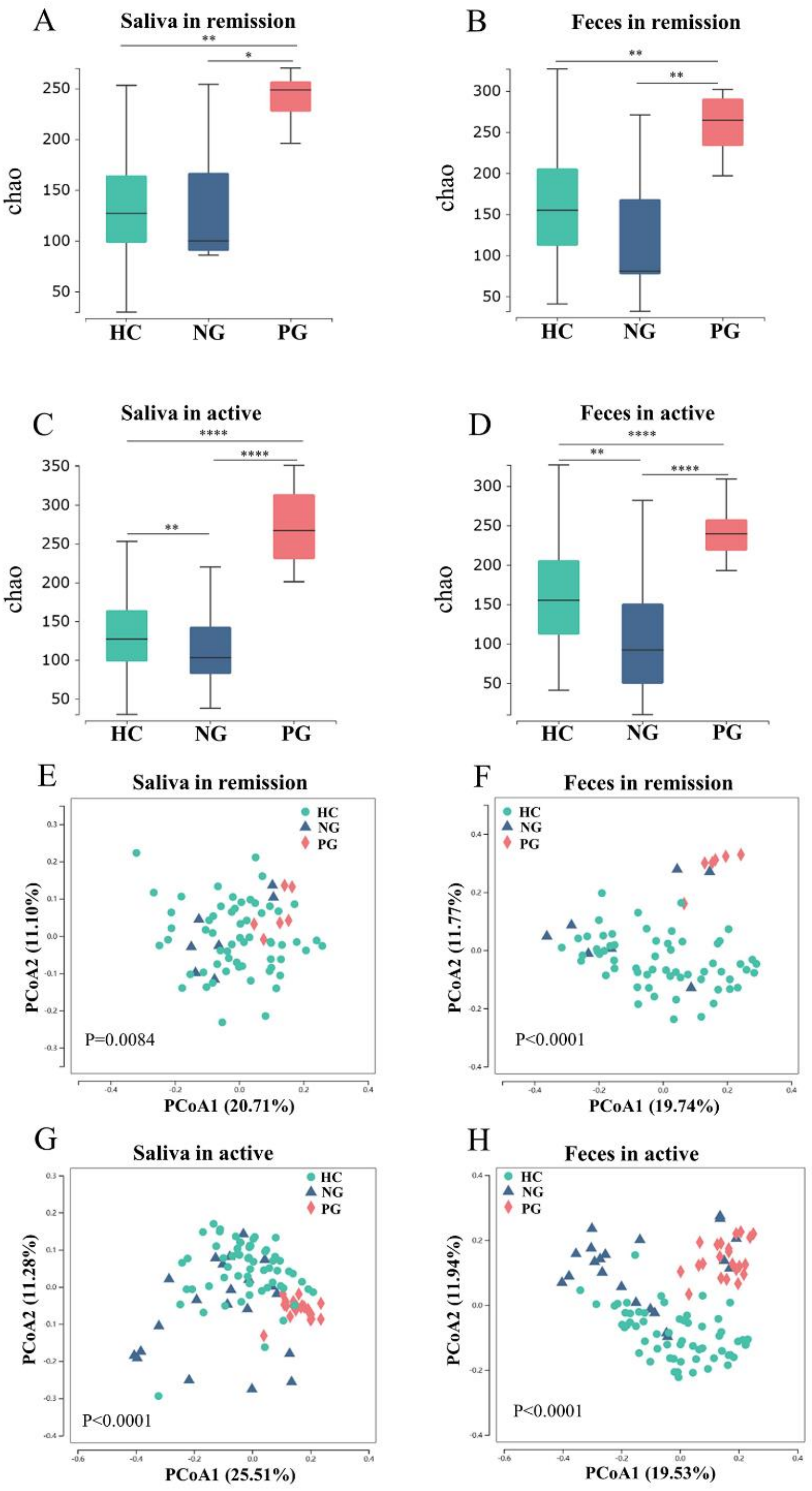

Figure S3. The diversities of oral and gut microbiota in active and quiescent CD. (A and B) The α-diversities of salivary and fecal microbiota in quiescent CD. (C and D) The α-diversities of salivary and fecal microbiota in active CD. (E and F) The β-diversities of salivary and fecal microbiota in quiescent CD. (G and H) The β-diversities of salivary and fecal microbiota in active CD. CD, Crohn's disease

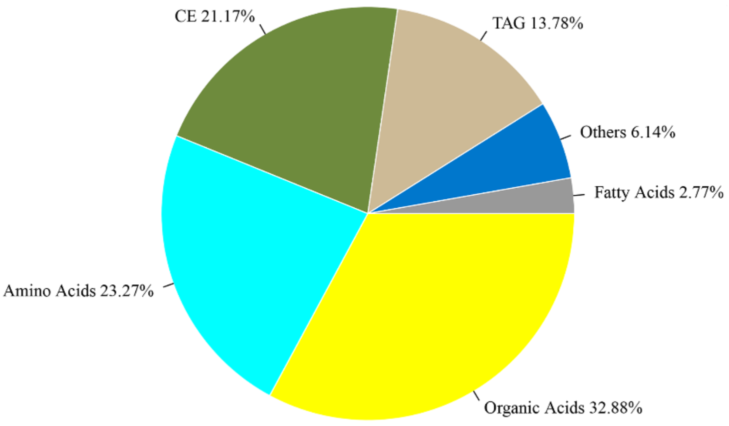

Figure S4. Pie chart showing the average abundance of metabolites across all samples.

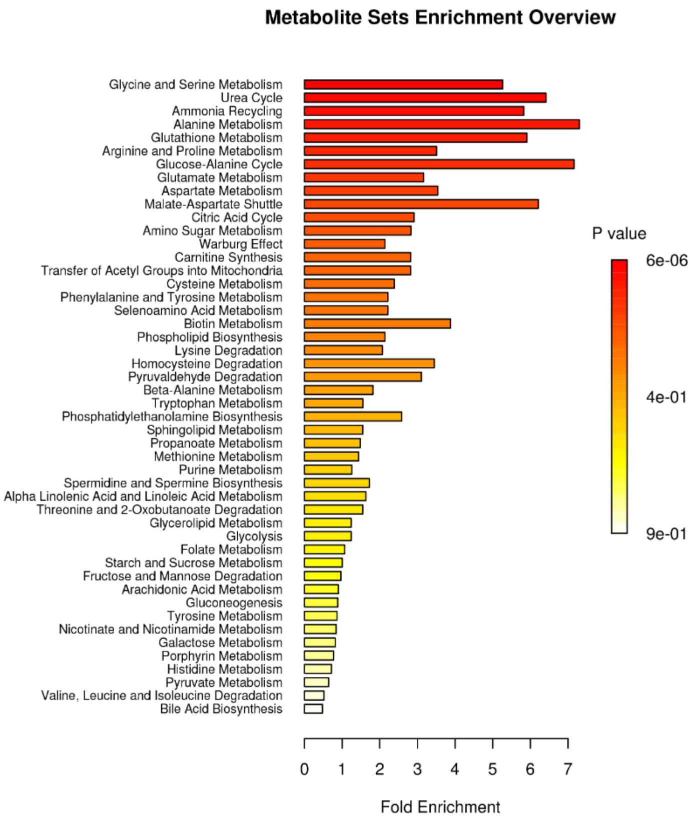

Figure S5. Pathway Enrichment Analysis Barplot Using Pathway-associated metabolite sets (Pathway-associated metabolite sets [SMPDB]).
